# Production and Evaluation the Pharmacokinetic Effects of Probiotic Mouthwashes and Their Impacts on Periopathogenic Bacteria in Vitro: Pharmacokinetic Effects of Probiotic Mouthwashes on Periopathogenic Bacteria

**DOI:** 10.31661/gmj.v13iSP1.3655

**Published:** 2024-12-31

**Authors:** Alireza Etezadinia, Sahel Shahrestani, Maryam Kakoienejad, Nima Naddaf Pour

**Affiliations:** ^1^ Department of Periodontology, Shahed Dental School, Shahed University, Tehran, Iran; ^2^ Operative Dentistry Department, Shahed Dental School, Shahed University, Tehran, Iran; ^3^ Department of Periodontology, Faculty of Dental, Tehran University of Medical Sciences, Islamic Azad University, Tehran, Iran

**Keywords:** Lactobacillus Reuteri, Lactobacillus Rhamnosus, Aggregatibacter Actinomycetemcomitans, Porphyromonas Gingivalis Mouthwash

## Abstract

**Background:**

In this study, the pharmacokinetic effects of Lactobacillus rhamnosus (L.rh) and Lactobacillus reuteri (L.r) mouthwashes were investigated and the effects of these two bacteria on the Aggregatibacter actinomycetemcomitans (A.a) and Purofiromonas gingivalis (P.g) were compared. The results indicated which of the following probiotics has the inhibitoriest effect on priopathogens.

**Materials and Methods:**

Two types of mouthwash containing two probiotics; L. reuteri and L. rhamnosus, were produced. To evaluate the pharmacokinetics of each of the probiotic strains in the mouthwashes, tests for hydrogen peroxide resistance, lysozyme resistance, quantitative calculation of organic acids, and disk diffusion were performed. The Antipathogenic test was also performed to determine the extent of growth inhibition of the mouthwashes against the periopathogens of Aggregatibacter actinomycetemcomitans and Porphyromonas gingivalis.

**Results:**

L. rhamnosus probiotic was more resistant to hydrogen peroxide but less resistant to lysozyme enzyme than L. reuteri. The production of organic acids after 72 hours of incubation at 37 ͦ C in the L. reuteri strain was significantly higher than the L. rhamnosus. The amount of growth inhibition zone formed by the periopathogenes was detected. Both strains of lactobacilli used in the mouthwashes had good resistance to antibiotics.

**Conclusion:**

L. reuteri had a higher resistance to the enzyme lysozyme. However, due to the higher production of organic acids and the possibility of its negative impact on the structure of tooth enamel, the use of this bacteria is not ultimately desirable to maintain oral hygiene. According to the data of this study, due to the high resistance of L. rhamnosus to hydrogen peroxide and antibiotics and its greater effect on periopathogenic strains, the use of this bacteria in comparison with L. reuteri in the laboratory environment has more advantages.

## Introduction

Today, periodontal disease is one of the most common diseases of the oral cavity, which leads to the loss of supporting structures around the teeth, including bone and periodontal fibers. Alteration in the balance of normal oral microflora and its transformation into periodontal bacteria is considered the main cause of periodontal disease, the initial manifestations of which appear as gingivitis, and if it progresses, it develops into periodontitis [1]. Accumulation of dental plaque due to poor oral hygiene, which covers the upper and lower areas of the gums, leads to "Increased green complex bacteria such as Aggregatibacter actinomycetemcomitans and Capnocytophage species, and red complexes such as Porphyromonas gingivalis, Tanerla furcitia and Terpenoma denticula [2]. These complexes cause oral complications such as bleeding during probing, pregnant tongue, bad breath, periodontal disease [3], and systemic problems including cardiovascular disease, premature birth, diabetes, etc. [4].

Aggregatibacter actinomycetemcomitans (A.a) is known as the main periopathogen of destructive diseases of local invasive periodontitis. The presence of this pathogen has been confirmed in the following cases: Chronic periodontal disease, bad breath, non-alcoholic fatty liver disease, rheumatoid arthritis, hypertension, cardiovascular disease, diabetes, low HDL blood, and neonatal weight loss [5][6][7][8].

Porphyromonas gingivalis is known as one of the main pathogens involved in chronic periodontitis. It has also been linked to systemic diseases such as coronary artery disease, diabetes and insulin resistance, oral and colorectal carcinoma, Alzheimer’s disease, neonatal weight loss, and bacterial lung infection [9][10][11].

Current treatments for periodontal disease include plaque control, antibiotic therapy, and periodontal and laser surgery [12][13]. Plaque control is done mechanically (brushing, flossing, scaling) and chemically (prescribing mouthwashes and detectors) [14]. Currently, the most common mouthwash to control plaque is chlorhexidine digluconate, which is used to control plaque in recurrent periodontal disease and after periodontal or oral surgery. Moreover, it has a great effect on gram-negative bacteria [15].

The appearance of bacteria resistant to antibiotic therapy has become a global problem and has led to the discovery of new ways to control this infectious disease [16]. So studies have been conducted on alternative strategies for the use of antibiotics, such as protease-inhibiting agents and bacterial tissue-destroying agents [17].

The use of probiotics has become more common in recent years. Probiotics are used to treat oral diseases such as caries, gingivitis, periodontitis, heartburn syndrome, dry mouth, and candidiasis [18]. In recent decades, many bacteria have been used as probiotic products, the most famous of which are strains belonging to the Lactobacillus family [19][20]. Various studies have shown the beneficial effects of Lactobacillus reuteri and Lactobacillus rhamnosus in the treatment of oral diseases; therefore, the same two lactobacilli were used in this study [21][22].

This study aimed to produce and evaluate the pharmacokinetic effects of Lactobacillus reuteri mouthwash with Lactobacillus rhamnosus mouthwash and also to compare the effects of these two types of mouthwash on A.actinomycetecomitans and P.gingivalis. In addition to examining the pharmacokinetic effects of these mouthwashes, we also found which probiotic mouthwashes are more effective on periopathogenic bacteria.

## Materials and Methods

### Preparation of Bacterial Target Strain

In this in vitro study, bacterial strains belonging to the Aggregatibacter actinomycetemcomitans and Porphyromonas gingivalis family were exposed to probiotic mouthwashes of Lactobacillus reuteri and Lactobacillus rhamnosus. For this purpose, the bacteria were purchased as a lyophilized powder from the Bank of Iran Microorganisms Center. Each vial of these bacteria (in aseptic conditions) was opened to add 0.3 to 0.5 of sterile physiologic saline (NaCl 0.09%). These vials were then incubated at 37 ͦC for 2-4 hours. Afterward, each of the vials with viable and homogenous bacterial strains was transferred to tubes with MRS broth and incubated at 37 ͦC for 24 hours. From each of these vials, sterile fildoplatin (Loop) culturing was performed in two plates of MRS Agar and they were incubated at 37 ͦC for 24-48 hours. From each of the plates, 3-4 colonies of bacteria were suspended in physiological serum to obtain 1-2 * 108 CFU/mL of each bacteria. A suspension of 0.5 McFarland turbidity was obtained from the bacteria strains (grown in MRS broth culture medium) in a solution of buffer saline phosphate and podmaltodextrin and shaked [23][24].

### Preparation of Probiotic Mouthwash

To prepare probiotic mouthwash, Lactobacillus reuteri and Lactobacillus rhamnosus bacteria were purchased as a lyophilized powder from the Bank of Iran Microorganisms Center. Each vial of these bacteria (in aseptic conditions) was opened and 0.3 to 0.5 of sterile physiologic saline (NaCl 0.09%) was added to it. These vials were incubated at 37 ͦC for 2-4 hours. Then, each of the vials with viable and homogenous bacterial strains was transferred to tubes with MRS broth and incubated at 37 ͦC for 24 hours. From each of these vials, sterile fildoplatin (Loop) culturing was performed in two plates of MRS agar and they were incubated at 37 ͦC for 24-48 hours. From each of the plates, 3-4 colonies of bacteria were suspended in physiological serum to obtain 1-2 * 108 CFU/mL of each bacteria. A suspension of 0.5 McFarland turbidity was obtained from the probiotic strains (grown in MRS broth culture medium) in a solution of buffer saline phosphate and podmaltodextrin and shaked. Probiotic mouthwash was obtained by mixing the suspensions of two strains of bacteria (50 mL of L.reuteri with 50 mL of L. rhamnosus) with a homogeny suspension of probiotic bacteria [25].

### Resistance to H2O2

11.33 ml of H2O2 solution (Sigma Aldrich Company) with a concentration of 30% was taken to 100 ml using distilled water to achieve a concentration of 1 M of H2O2. Broth culture medium was prepared according to the probiotic strains in mouthwashes with a volume of 10 ml. 10 μl, 25 μl, 50 μl, and 150 μl of 1 M H2O2 were added to the first 4 broth media, respectively, under sterile conditions, and no material was added to the fifth culture medium. Bacteria were counted in five inoculated media and then all of them were incubated at 37 °C for 24 hours. Bacterial colonies were counted from each medium at T6 and T24 [26].

### Lysozyme Resistance

Broth culture medium was prepared in accordance with the probiotic strains in the mouthwashes, with a volume of 10 ml. 1 mg, 2.5 mg, 5 mg, and 50 mg of lysozyme (Sigma Aldrich Company) were applied to the first 4 broth media, respectively, under standard laboratory conditions. No material was added to the fifth culture medium. After preparing the inoculated medium, probiotic bacteria were prepared for 24 hours under anaerobic conditions at 37 °C, and then an equivalent to 1% of the volume of the broth culture media (100 μl) of probiotic bacteria was introduced into all 5 media. These five media were inoculated, bacteria were counted, and all of these media were incubated at 37 °C for 24 hours. Each culture medium was counted at T6 and T24 [26].

### Quantitative Calculation of Organic Acids

Culture medium (PH=6.68) Skim milk (10%) was prepared.

The fresh culture was prepared from the frozen of lyophilized probiotic bacteria in MRS culture medium, after 24 hours Incubation, 1% of bacteria were suspended and inoculated into Skim milk culture medium (10%).

All media were incubated at 37 °C for 72 hours under anaerobic conditions containing 10% CO2 and 90% N2.

To determine the acidity of the Skim Milk culture medium, it was poured into Erlenmeyer and 5 drops of 1% phenolphthalein solution were added and the resulting solution was titrated with 0.1 N NaOH. When the color reached purple, the titration was stopped and then calculated to determine the acidity in milliliters according to the following formula [27]:


X = \frac{N \times 0.009}{M}

X: Percentage of acidity

N: NaOH (0.1N) ml

M: Sample weight (gr)

Growth Inhibition Zone Determination (Antipathogenic Test)

The primary culture of the aggregate bacteria Actinomycetem comitans and Porphyromonas gingivalis was done in MRS broth under anaerobic conditions for 24 hours. The 0.5 McFarland suspension was prepared from the mentioned priopathogenic strains. Using a swap, 50 microliters of the content of each of each of the probiotic mouthwashes were also cultured separately and linearly in the middle of the BHI agar culture medium, anaerobically and for 24 hours. The next day, after the growth of probiotic bacteria in the middle part of the plates, the priopathogens prepared with the standard amount of 0.5 McFarland’s were transferred to the BHI agar culture medium by swap and were cultured in a vertical line. In this way, priopathogenic bacteria were exposed to the probiotic strains in the mouthwashes for 24 hours in anaerobic conditions and at a temperature of 37 ͦ C. Then, the non-growth zone obtained from the probiotic strains was determined using calipers [28]. The standard crude disk containing chlorhexidine was also placed as a positive control in the culture medium of the mentioned pathogens [29].

### Disk Diffusion

Each 0.5 McFarland suspension probiotic mouthwash was cultured separately on MRS agar on all sides to cover the entire surface of the plate. The plates were placed at room temperature for 5-10 minutes to absorb moisture. Antibiotic disks were placed with the help of sterile tweezers next to the flame and under the microbiology hood with a distance of 20 mm from the edge of the plate and 25 mm from each other on the surface of the plate. The antibiotic discs included: Ceftriaxone (30 micrograms), metronidazole (30 micrograms), penicillin (10 micrograms), amoxicillin clavulanate (10.20 micrograms), amoxicillin (25 micrograms), erythromycin (15 micrograms), vancomycin (30 micrograms), azithromycin (15 micrograms), ciprofloxacin (5 micrograms). To prevent perspiration, the plates were inverted and transferred to an incubator at 37 °C. The results were evaluated after 24 hours. The measurement step was done without opening the plate, only from the back of the plate with a ruler or caliper. Standard tables provided by CLSI were used to interpret the results. Using these tables and the diameter of the growth inhibition zone of each antimicrobial drug, the bacteria were sorted into resistant, semi-susceptible, and susceptible categories [30].

### Statistical Analysis

Mean and standard deviation were used for quantitative data, and number, percentage, and bar graph were used for qualitative data. Two-way ANOVA and RM-ANOVA tests, at a significance level of 0.05, were also used to evaluate the mean differences between various groups and time intervals. IBM SPSS Statistics for Windows, Version 24.0, was used for all statistical analyses. The basis for choosing the number of samples in this study was the number of patients referred to the dental clinic of the university.

## Results

**Figure-1:**
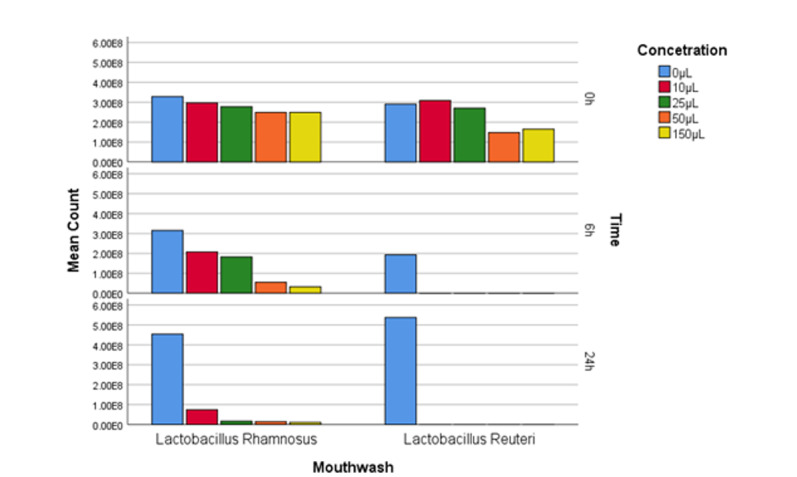
Figure 1. Probiotic mouthwashes colony count mouthwashes in hydrogen peroxide resistance test at different concentrations (regardless of time intervals)

RM-ANOVA test was performed to evaluate the resistance of hydrogen peroxide at different concentrations and time intervals. The results indicate the Lactobacillus rhamnosus strain has had more growth in the presence of different concentrations of H2O2 enzyme and at different time intervals, While Reuteri lactobacilli were considerably more sensitive to H2O2 than Rhamnosus lactobacilli, as the RM-ANOVA test reports a significant difference between these two probiotic strains, (P<0.01, Figure-1).

**Figure-2:**
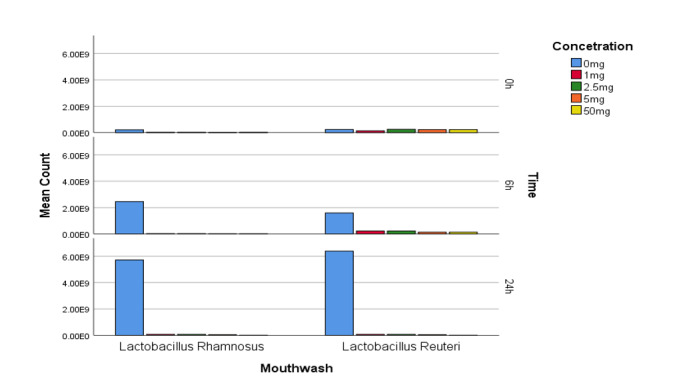
Figure 2. Probiotic mouthwashes colony count in lysozyme resistance testing at different concentrations and times

Regarding the lysozyme resistance test, according to the statistical analysis, it can be concluded that at different concentrations of lysozyme enzyme and at different time intervals, the Lactobacillus reuteri strain has had more growth. Although Lactobacillus rhamnosus is more sensitive to lysozyme than reuteri. This difference is statistically significant, (P<0.01, Figure-2).

According to the findings of the RM-ANOVA test for quantitative calculation of organic acids, During T24, the amount of acid produced by Lactobacillus rhamnosus strain was higher, while during T48 and T72, the amount of acid produced by Lactobacillus roteri strain was higher. This difference is statistically significant (P<0.01). Table-1 shows the results of the acidity test.

**Figure-3:**
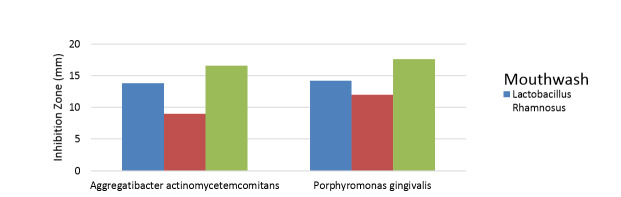
Figure 3. Investigation of growth inhibition zone of probiotic mouthwashes in anti-pathogenic test (growth inhibition zone is in millimeters)

In the study of the zone of non-growth of mouthwash bacteria in the anti-pathogenic test, according to the two-way ANOVA, it can be concluded that inhibiting P.G. strain is most effective by chlorhexidine mouthwash followed by Lactobacillus rhamnosus probiotic mouthwash. The difference in growth inhibition zone applied by mouthwashes is statistically significant, (P<0.01). Chlorhexidine mouthwash is proved to be the most effective in inhibiting A.a strain, followed by Lactobacillus rhamnosus probiotic mouthwash. The difference in growth inhibition halo applied by mouthwashes is statistically significant (P<0.01). In control of both pathogens, Lactobacillus reuteri probiotic mouthwash has had the lowest growth inhibition zone, which is significantly lower than the other two types of mouthwash, (P<0.01, Figure-3).

In the disk diffusion resistance test in different antibiotics according to the CLSI table, it can be concluded that all lactobacilli strains have sufficient resistance in certain concentrations of antibiogram disks. Concerning Lactobacillus rhamnosus however, it is only relatively sensitive to the antibiotic ciprofloxacin, (Table-2).

## Discussion

**Table:** Table1. Evaluation of Probiotic Mouthwashes in the Quantitative Calculation Test of Organic acids

Type of mouthwash	Time	Number	Minimum	Maximum	Mean	Standard deviation
	24 h	5	0.30	0.33	0.3128	0.00960
Lactobacillus Rhamnosus	48 h	5	0.30	0.40	0.3670	0.03899
	72 h	5	0.64	0.66	0.6466	0.00882
	24 h	5	0.28	0.32	0.2986	0.01448
Lactobacillus Reuteri	48 h	5	0.35	0.41	0.3824	0.02215
	72 h	5	0.81	0.88	0.8394	0.02563

**Table:** Table2. Evaluation of Bacterial Resistance in Disk Diffusion Resistance Test in Different Antibiotics

Type of mouthwash	Type of antibiotic	Resistance status	Number	Percentage
	ceftriaxone	Resistant	5	100.0
	Metronidazole	Resistant	5	100.0
	Penicillin	Resistant	5	100.0
	Amoxicillin clavuene	Resistant	5	100.0
Lactobacillus Rhamnosus	Amoxicillin	Resistant	5	100.0
	Erythromycin	Resistant	5	100.0
	Vancomycin	Resistant	5	100.0
	Azithromycin	Resistant	5	100.0
	Ciprofloxacin	Semi-sensitive	5	100.0
	ceftriaxone	Resistant	5	100.0
	Metronidazole	Resistant	5	100.0
	Penicillin	Resistant	5	100.0
	Amoxicillin clavuene	Resistant	5	100.0
Lactobacillus Reuteri	Amoxicillin	Resistant	5	100.0
	Erythromycin	Resistant	5	100.0
	Vancomycin	Resistant	5	100.0
	Azithromycin	Resistant	5	100.0
	Ciprofloxacin	Resistant	5	100.0

Studies have shown that mechanical solutions such as brushing or SRP alone are not enough to reduce bacterial volume and prevent oral diseases [31][13]. For this reason, along with mechanical methods, antibiotics were also used in the treatment of periodontal diseases [32][33]. With the beginning of the spread of antibiotic resistance, the use of antibiotics was limited [34][35], researches were looking for alternatives to antibiotics. In this way, even the use of substances with antimicrobial properties or protease inhibitors was suggested [36][37]. Nowadays, it is tried to use targeted treatments that directly affect periodontal strains instead of non-specific treatments to control plaque [26]. Probiotic strains can play an important role in oral and dental health and prevent oral diseases such as gingivitis, periodontitis and caries by inhibiting pathogenic strains [39][40]. The guidelines published by the WHO organization have stated the essential properties of probiotics, based on these instructions, experiments have been designed to measure these characteristics [12]. Considering the use of two probiotic strains of Lactobacillus roteri and Lactobacillus rhamnosus in different commercial products, this study was conducted with the aim of comparing the pharmacokinetic properties of these two probiotic strains with each other and their effect on two important pathogen strains involved in periodontal diseases.

Studies have already revealed that mechanical solutions, such as brushing or SRP, are not enough to reduce bacterial volume and prevent oral diseases by themselves [13]. Consequently, in some cases, antibiotic therapy is used to treat periodontal disease along with conventional mechanical methods [14]. But due to the spread of bacterial resistance to antibiotics, their prescription has been limited [17].

Therefore, researchers have been seeking alternative solutions to the use of antibiotics, such as the use of protease-inhibiting substances and bacterial tissue-destroying factors [18]. Today, instead of non-specific treatments such as mechanical plaque control solutions, targeted therapies that directly affect pathogens are tried. One of the most important of these methods is the use of compounds containing probiotic strains, the use of which has become more common in recent years. Out of the many bacteria used as probiotic products, strains belonging to the Lactobacillus family are of great importance [20][21]. Among the effective lactobacilli in the treatment of oral diseases are Lactobacillus reuteri and Lactobacillus rhamnosus. The effects of the mentioned bacteria on oral pathogenic bacteria have been proposed in various articles. The use of these two strains in different commercial products has also become common [22][41][42].

Due to the use of these two probiotic strains in different commercial products, this study aimed to investigate the pharmacokinetics of these two probiotic strains with factors affecting oral microorganisms in vitro. In addition, the effects of these two probiotic strains on the two pathogenic strains of Porphyromonas gingivalis and Aggregatibacter actinomycetemcomitans were determined and compared. In 2011, Teanpaisan et al., at the Center of Epidemiological Research and Oral Diseases in Thailand, examined the reducing effect of probiotic lactobacilli on oral pathogens in vitro. Samples of oral pathogens were collected from 2 to 5-year-old children participating in the experiment. 10 families of Lactobacilli, including Lactobacillus fermentum, Lactobacillus rhamnosus, Lactobacillus salivaris, Lactobacillus vaginalis, Lactobacillus gasseri, Lactobacillus mucosa, Lactobacillus casei, Lactobacillus paracasei, Lactobacillus eris, and Lactobacillus plantarum, were used in the experiment. Then the growth inhibitory effect of pathogenic bacteria by lactobacilli was measured. The results illustrated that probiotics of Lactobacillus paracasei, Lactobacillus casei, Lactobacillus salivarius, Lactobacillus rhamnosus, Lactobacillus plantarum against Streptococcus sorbinus, Streptococcus mutans, Porphyromonas gingivalis, and Aggregatibacter actinomycetemcomitans have a strong inhibitory effect [29].

In a study by Bosch et al., experiments were performed on 46 strains of Lactobacillus bacteria isolated from the mouth and feces, most of which belonged to the Lactobacillus family. Their analyses of the autoaggregation, coaggregation, H2O2, and lysozyme resistance tests proved that there was no difference between the strains isolated from the mouth and feces. Furthermore, antipathogenic tests were performed on F.nucleatum, T.denticola, P.gingivalis, and S.mutans strains which inhibited 11 probiotic strains, one pathogen; 8 probiotic strains, 2 pathogen strains; 15 probiotic strains, 3 pathogen strains; and 11 probiotic strains, 4 pathogen strains. P.G. was reported as the least inhibited strain, while the most inhibited one was P.denticola. Finally, in this study, according to the test results, it was claimed that 7 strains had better results than the other strains. Acid production and antibiotic susceptibility tests have been performed on this strain. All strains were sensitive to antibiotics. In the acid production experiment, oral strains produced less acid production [26].

Madhwani and McBain investigated the effect of lactobacillus reuteri on oral biofilm in vitro. In this study, immature plaques in the form of designed models of hydroxyapatite discs and mature interconnected plaques in fixed-depth film fermenters (CDFFs) were exposed to Lactobacillus reuteri probiotics. L. reuteri strains were evaluated in microbial composition using live differential counting. Strains in CDFF plaque were identified using qPCR and PCR-DGGE. The dose of Lactobacillus reuteri in immature plaques significantly increased. There was also an increase in gram-negative anaerobes and other lactobacilli in immature plaques in both biofilm and planktonic phases. The rise in the number of streptococci occurred only in the planktonic phase, which was not associated with a decrease in the pH environment. In adult CDFF plaques, the differential culture showed that while there was a significant increase in the number of Lactobacillus, the number of bacteria in the other groups and the pH of the medium did not change significantly. The lack of inhibitory effect of L. reuteri strain in both tested dental plaque systems was supported by no contradiction in binary antagonism assay. Finally, it was concluded that most compound changes occur in immature plaques and the addition of the Lactobacillus reuteri strain caused a significant increase in streptococci and gram-negative bacteria [43].

H2O2 and lysozyme resistance tests indicated resistance of the strain in probiotic mouthwash to oral conditions. More precisely, the aim was to investigate the effect of lysozyme and saliva H2O2 on probiotic strains in laboratory-simulated conditions. The concentrations used in the test were even several times higher than the concentrations in the oral environment to measure the resistance of the strains to more difficult conditions [26]. A comparison of probiotic growth rate in and Lactobacillus rhamnosus mouthwashes showed that the Lactobacillus reuteri strain was more resistant to lysozyme. It was also found that Lactobacillus rhamnosus strain is more resistant to hydrogen peroxide. In the quantitative calculation of organic acids, it was concluded that the higher the acid production, the more effective the acid-sensitive bacterial strains, such as the P.G and P.I periopathogenic strains. Their function was also proved to be impaired [44][45]. However, it is important to note that lowering the pH of the mouth demineralizes the enamel and creates favorable conditions for the growth of cariogenic bacteria. Therefore, a higher production of organic acids of probiotic strains in mouthwash may be more desirable [26]. In our study, it was found that although in the first 24 and 48 hours of testing, the lactobacillus reuteri and rhamnosus strains in mouthwashes produced approximately the same number of organic acids. But 72 hours after testing, the Lactobacillus reuteri strain produced more organic acids, which makes it less desirable. Therefore, Lactobacillus rhamnosus is a more beneficial strain in this regard.

In the diffusion disk test, it was found that more resistance of the strain according to the CLSI table causes less concern about the simultaneous use of antibiotics and probiotic mouthwash (). It is important to note that probiotic strains can transmit antibiotic-resistance genes [26][30]. The present study found that both probiotics used in mouthwashes have the desired minimum resistance (mentioned in the MIC test) against antibiotic discs with the above-mentioned concentrations. Other tests should be carried out to evaluate the maximum resistance of these strains to antibiotics.

The antipathogenic test, which is one of our major tests, showed the degree of inhibition of A.a and P.G. periopathogenic strains by probiotic strains in mouthwashes or their by-products. Products such as acid have been shown to inhibit the growth of strains such as P.G. that are sensitive to acidic compounds. Of course, this is only one of the mechanisms of inhibition of pathogenic strains by probiotic strains, and other mechanisms remain unknown [44][45].

This research demonstrated that the Lactobacillus rhamnosus mouthwash had more growth inhibitory properties against the two above-mentioned periopathogens than the Lactobacillus reuteri mouthwash. However, both probiotic mouthwashes had less inhibitory effect than the standard chlorhexidine treatment.

## Conclusion

According to the present study, it can be concluded that, on the whole, the Lactobacillus Rhamenosus mouthwash is more desirable than the Lactobacillus reuteri mouthwash. However, it is important to note that the oral environment is a complex polymicrobial conditions and the laboratory setting cannot be generalized to the oral environment. Therefore, to confirm the findings, it is recommended to use both types of mouthwash clinically to prove the effects of the Lactobacillus strains in them.

## Conflict of Interest

None.
